# A Smart Strategy to Improve *t*-Resveratrol Production in Grapevine Cells Treated with Cyclodextrin Polymers Coated with Magnetic Nanoparticles

**DOI:** 10.3390/polym12040991

**Published:** 2020-04-24

**Authors:** Lorena Almagro, Alicia De Gea-Abellán, María Isabel Rodríguez-López, Estrella Núñez-Delicado, José Antonio Gabaldón, María Angeles Pedreño

**Affiliations:** 1 Departamento de Biología Vegetal, Facultad de Biología, Universidad de Murcia, Campus de Espinardo, E-30100 Murcia, Spain; aliciadegeaabellan@gmail.com (A.D.G.-A.); mpedreno@um.es (M.A.P.); 2 Departamento de Tecnología de la Alimentación y Nutrición, Universidad Católica San Antonio de Murcia, Campus de los Jerónimos, E-30107 Murcia, Spain; mirodriguez@ucam.edu (M.I.R.-L.); enunez@ucam.edu (E.N.-D.); jagabaldon@ucam.edu (J.A.G.)

**Keywords:** carboxymethyl-β-cyclodextrin polymer-coated magnetic nanoparticles, hydroxypropyl-β-cyclodextrin polymer coated magnetic nanoparticles, methyl jasmonate, *t*-resveratrol, *Vitis vinifera* suspension-cultured cells

## Abstract

One of the most successfully procedures used to increase the production of *t*-resveratrol in *Vitis vinifera* suspension-cultured cells is the application of cyclodextrins (CDs) and methyl jasmonate (MJ) as elicitors. In particular, β-CDs are characterized by their chemical structure which makes them special, not only by acting as elicitors, but also because they are compounds capable of trapping high added-value hydrophobic molecules such as *t*-resveratrol. However, the use of β-CDs as elicitors increases the production costs of this compound, making their industrial exploitation economically unfeasible. Therefore, the development of β-CDs recovery strategies is necessary to provide a viable solution to their industrial use. In this work, carboxymethylated and hydroxypropylated β-CDs have been used to form polymers using epichlorohydrin (EPI) as a cross-linking agent. The polymers were coated to Fe_3_O_4_ nanoparticles and were jointly used with MJ to elicit *V. vinifera* suspension-cultured cells. Once elicitation experiments were finished, a magnet easily allowed the recovery of polymers, and *t*-resveratrol was extracted from them by using ethyl acetate. The results indicated that the production of *t*-resveratrol in the presence of free carboxymethyl-β-CDs was much lower than that found in the presence of carboxymethyl-β-cyclodextrins-EPI polymer coated magnetic nanoparticles. In addition, the maximal levels of *t*-resveratrol were found at 168 h of elicitation in the presence of 15 g/L hydroxypropyl-β-CDs polymer coated magnetic nanoparticles and MJ, and non-*t*-resveratrol was found in the extracellular medium, indicating that all the *t*-resveratrol produced by the cells and secreted into the culture medium was trapped by the polymer and extracted from it. This work also showed that polymers can be regenerated and reused during three cycles of continuous elicitation since the induction and adsorption capacity of hydroxypropyl-β-CDs polymer-coated magnetic nanoparticles after these cycles of elicitation remained high, allowing high concentrations of *t*-resveratrol to be obtained.

## 1. Introduction

The high nutritional, medicinal and economic value of the secondary metabolites that plants synthesize makes these compounds of great importance for cosmetic, food and pharmaceutical industries [1]. These industries try to find production techniques to obtain secondary metabolites that are reproducible, simple and have low economic costs. Currently, the production of secondary metabolites can be carried out by extraction from plant raw material, by chemical synthesis and from plant in vitro cultures [2]. In fact, plant in vitro cultures have the greatest advantages since the extracts obtained from them are homogeneous, and they have a growth in sterile conditions and indefinite maintenance, regardless of seasonal and weather conditions [2,3]. However, when the production of these metabolites wants to be extrapolated at the industrial level, it is necessary to optimize different parameters, and make a successful selection of elicitors in order to increase the production of a particular metabolite. Of the most successfully used elicitors to increase the production of *t*-resveratrol in *Vitis vinifera* cell cultures, cyclodextrins (CDs) and methyl jasmonate (MJ) stand out [4,5,6,7]. Jasmonic acid and its most active derivative, MJ, are known to be involved in several physiological processes in plants such as growth and development, and, more particularly, in the mediation of plant defence responses against abiotic and biotic stresses [8]. In addition, these types of elicitors have been widely used for elicitation experiments by using plant in vitro cultures [9]. In fact, MJ is the most commonly used elicitor, and it has a strong effect on the production of plant secondary metabolites in plant in vitro cultures [9,10]. Giri and Zaheer [9] conducted an extensive review in which they indicated that the three main elicitors used for the production of secondary metabolites were MJ, salicylic acid and jasmonic acid (approximately 60%, 15% and 10% of reports, respectively).

Likewise, CDs are a group of naturally occurring cyclic oligosaccharides with six, seven or eight glucose residues linked by α-(1,4)-glycosidic bonds in a shaped cylinder structure and named as α-, β- and γ-CDs, respectively [11]. They are obtained from starch enzymatic degradation by the action of a group of amylases such as cyclodextrinases or glycosyltransferases. The rims of the surrounding walls are hydrophilic, while the hydrophobic central cavity of these molecules forms inclusion complexes with a wide range of organic and inorganic compounds, considerably increasing the solubility, stability and bioavailability of the guest molecule [12]. Among the modified-cyclodextrins, 2-hydroxypropylated and methylated β-cyclodextrins are produced at industrial scale because they are considered useful carriers of antitumor- and immuno-regulatory drugs, and they can also be used as additives for the food industry [13]. It is also important to highlight the use of CDs for increasing the production of bioactive compounds from plant in vitro cultures because of CD’s ability to act as “hosts” of bioactive compounds favouring their accumulation in the aqueous culture medium [14,15]. In fact, the treatment of plant in vitro cultures with CDs and their derivatives increases the production of secondary metabolites such as *t*-resveratrol and other antioxidant compounds, enhancing the capability of plant in vitro cultures to produce high levels of different bioactive compounds which have human health beneficial properties [16,17]. However, the use of CDs increases production costs, making their industrial exploitation economically unfeasible. Therefore, the development of recovery strategies for these molecules is necessary in order to give a viable solution to their industrial use.

On the other hand, magnetic iron oxide nanoparticles have several applications such as cell labelling, drug delivery and molecular imaging, owing to their magnetic property and biocompatibility [18]. In fact, different strategies have been carried out to use non-toxic magnetic nanomaterial polymers as targets in cancer cells [19]. The biocompatibility of iron oxide nanomaterials with cells and tissues is increased when they are coated with polymers such as dextrans [20]. These polymers have a high affinity for iron oxide and low toxicity, and the bioactive compounds are either electrostatically bound or covalently conjugated with the outer surface of the polymer [21]. Due to CD hydrophobic cavity, making it able to form inclusion complexes with bioactive compounds of appropriate size, and a hydrophilic outer rim which makes them soluble in water, magnetic nanoparticles have been grafted with CDs, which are used as carriers. In fact, Lungoci et al. [22] generated nanocarriers composed of magnetite nanoparticles which formed inclusion complexes between anionic sulfobutylether-β-CDs and protocatechuic acid for active drug delivery. Moreover, Gong et al. [23] showed that carboxymethyl- and hydroxypropyl-β-CDs polymers modified with magnetic particles Fe_3_O_4_ were able to extract rutin, a flavonoid glycoside able to increase the resistance of the capillaries and dissolve the fat invading the liver.

In this work, carboxymethylated and hydroxypropylated β-CDs have been used to form polymers using epichlorohydrin (EPI) as a cross-linking agent, and the polymers were coated with Fe_3_O_4_ nanoparticles and jointly used with MJ to elicit *V. vinifera* suspension-cultured cells in order to increase the production of *t*-resveratrol, and these polymeric CDs coated with magnetic nanoparticles were easily recovered to give a viable solution to their industrial use.

## 2. Materials and Methods

### 2.1. Chemicals

The MJ and plant culture media used were from Duchefa (Valencia, Spain). Carboxymethyl β-cyclodextrin (CM-β-CDs) sodium salt (substitution degree: 3.5, 95% purity) and 2-hydroxypropyl-β-cyclodextrin (HP-β-CDs) (substitution degree: 4.5) were purchased from Cyclolab (Budapest, Hungary). Iron (III) chloride hexahydrate (FeCl_3_·6H_2_O, 99%), iron (II) chloride tetrahydrate (FeCl_2_·4H_2_O, 99%), EPI (99%), sodium borohydride (98%), ammonium hydroxide (28–30%) and sodium hydroxide (98%) were purchased from Sigma-Aldrich (Madrid, Spain).

### 2.2. Plant Material

*Vitis vinifera cv*. Monastrell calli were established and maintained as previously described by Calderon et al. [24]. Calli were subcultured every 30 days on solid culture medium as described previously by Belchí-Navarro et al. [6]. To obtain suspension-cultured cells, 20 g fresh weight (FW) cells were transferred into 250 mL flasks containing 100 mL of liquid culture medium, and they were subcultured every 15 days.

### 2.3. Synthesis of CD Polymers Coated with Magnetic Nanoparticles

Firstly, bare Fe_3_O_4_ nanoparticles were synthesized using the co-precipitation method described by Kandpal et al. [25], with slight modifications. Briefly, Fe (III) and Fe (II) salts in a molar ratio of 3:1, were dissolved in 100 mL water. Then ammonium hydroxide (30 mL) was added dropwise to the mixture until its pH was raised to get a homogenized black-coloured solution. After that, the solution was stirred vigorously at 80 °C for 30 min and centrifuged at 4000 rpm for 10 min. Magnetic nanoparticles were separated by using a permanent magnet, washed with distilled water:ethanol (1:1) several times and subsequently dried in a vacuum oven at 45 °C overnight.

For the synthesis of CM-β-CDs-EPI and HP-β-CDs-EPI polymers coated Fe_3_O_4_ nanoparticles, a modification of the method described by Badruddoza et al. was used [26]. For that, 60 mg of sodium borohydride was mixed with 24 g of each CD (CM-β-CDs- or HP- β-CDs) in 24 mL of water. After stirring for 10 min at 50 °C, 26 mL of sodium hydroxide (40%) was added to the solution, which was stirred for 5 min. Then, the Fe_3_O_4_ nanoparticles were incorporated in the same proportion as the CD (24 g) to the solution. Afterwards, 264 g of EPI were added dropwise, ant this mixture was stirred for 6 h at 50 °C until the solution became a polymer. Then, the polymer was subjected to successive washing steps with distilled water:acetone (2:1), and dried in an oven overnight at 60 °C.

Field emission scanning electron microscope (FE-SEM) images of CD polymers coated with magnetic nanoparticles were obtained on an Apreo S field emission scanning electron microanalyzer equipped with EDX detector (Thermo Scientific Brno, Brno, Czech Republic). The energy dispersive X-ray spectroscopy mapping was performed at an accelerating voltage of 20 kV.

### 2.4. Vitis vinifera cv. Monastrell Cell Culture Treatments

**Figure 1:**
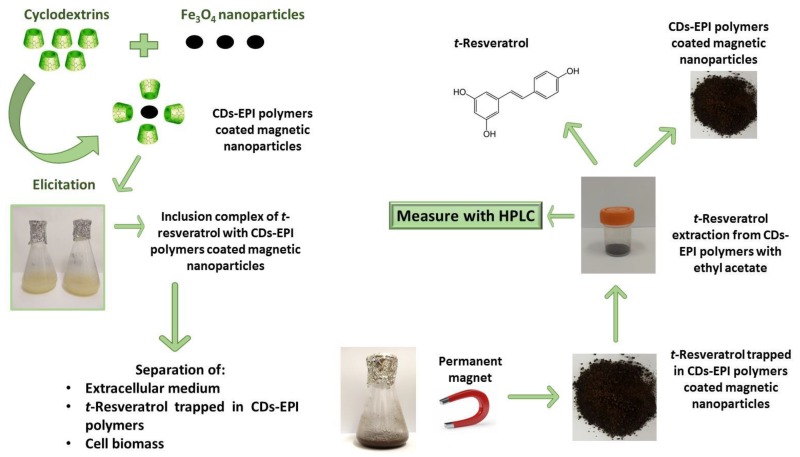
Scheme of the operation way of β-cyclodextrins-epichlorohydrin (CDs-EPI) polymers coated magnetic nanoparticles and their use as both inducers and adsorbents of *t*-resveratrol produced by *Vitis vinifera* cells treated with 100 µM methyl jasmonate (MJ).

The elicitation experiments were performed in triplicate using 15 days old suspension-cultured cells of *V. vinifera*. For this, 4 g FW cells were added to 20 mL of liquid culture medium as described by Belchí-Navarro et al. [6]. The first experiments consisted of the elicitation of *V. vinifera* suspension-cultured cells with different concentrations (0.5, 5, 7.5, 10 and 15 g/L) of free CM-β-CDs, or with the polymer coated with magnetic nanoparticles (CM-β-CDs-EPI-MN) in the presence of 100 µM MJ for 144 h of treatment at 25° C in the dark in a rotary shaker (110 rpm). In addition, the same conditions were used in *V. vinifera* suspension-cultured cells treated with free HP-β-CDs or forming a polymer with EPI coated with magnetic nanoparticles (HP-β-CD-EPI-MN) in the presence of 100 µM methyl jasmonate (MJ) for 144 h of treatment (Figure 1). After selecting the type and concentration of CDs that produced the highest levels of *t*-resveratrol, the time course of the production of *t*-resveratrol in *V. vinifera* suspension-cultured cells elicited with 100 µM MJ and 15 g/L HP-β-CD-EPI-MN was carried out. For this, *t*-resveratrol production from grapevine cells was periodically analysed at 24, 72, 144, 168, 216, 240 and 312 h of treatment. Finally, the variation in *t*-resveratrol production from grapevine cells was carried out through three continuous elicitation cycles (168 h each) using the same HP-β-CD-EPI-MN polymer but changing grapevine cells which, in turn, were elicited with the reused HP-β-CD-EPI-MN polymer in combination with 100 µM MJ. The final cell density used in all elicitation experiments was 200 g FW/L. Analysis of grapevine cell growth was performed in triplicate at the end of each experiment after each elicitor treatment. Cell growth of *V. vinifera* suspension-cultured cells were determined as previously described by Belchí-Navarro et al. [6].

Cell viability was measured by using two fluorescent probes, fluorescein diacetate for detecting living cells, and propidium iodide for identifying dead cells [6]. A fluorescence microscope (Leica Microsystems Inc. Wetzlar, Germany) was used to detect the fluorescence in grapevine cells.

### 2.5. Extraction and Quantification of t-Resveratrol

Once elicitation experiments were finished, the following common procedures were carried out. In order to achieve cell separation from the culture medium and the polymer-coated Fe_3_O_4_ nanoparticles, a magnet was used (Figure 1). For this, the magnet was placed on the bottom surface of the flask where the CM-β-CD-EPI-MN or HP-β-CD-EPI-MN polymers were retained, and then *V. vinifera* suspension-cultured cells were filtered by using a borosilicate glass funnel coupled to a Büchner flask. The magnet ensured that the polymers of CM-β-CD-EPI-MN or HP-β-CD-EPI-MN remained inside the flask while the cells remained in the borosilicate glass funnel, and the extracellular medium was recovered in the Büchner flask. Once the three components were separated, the magnetic polymers were washed three times with distilled water to remove cell debris or the extracellular medium. Then, *t*-resveratrol was extracted from the CM-β-CD-EPI-MN or HP-β-CD-EPI-MN with ethyl acetate (1:10 *w/v*), by stirring with a magnetic stirrer for 40 min (Figure 1). This extraction process was repeated three times. Afterwards, the ethyl acetate was collected and evaporated at 40 °C in vacuum; the residue was dissolved in methanol and analysed by using a HPLC-DAD (Waters 600E, Waters 996, Milford, Massachusetts, USA), as described by Belchí-Navarro et al. [6]. Thus, 20 µL of diluted and filtered (Anopore 0.2 µm) samples were analysed in a HPLC-DAD (Waters 600E, Waters 996) using a Spherisorb ODS2 C-18 column (250 × 4.6 mm, 5 µm). The column was eluted at 1 mL/min in gradient mode: solvent A, 0.05% trifluoroacetic acid; solvent B, 0.05% trifluoroacetic acid in methanol/acetonitrile 60:40 *v/v*. *t*-Resveratrol was identified at 306 nm and quantified by comparison with commercial *t*-resveratrol of >99% purity (Sigma Aldrich, Madrid, Spain), which was used as standard.

### 2.6. Preliminary Tests for Absorption-Desorption of t-Resveratrol from the Magnetic Polymers

Commercial *t*-resveratrol was loaded into magnetic polymers by means of the formation of inclusion complexes. For this, 150 mg of HP-β-CD-EPI-MN polymer was dispersed in 25 mL of distilled water which contained a commercial *t*-resveratrol concentration of 50 mg/L (219 µM). The *t*-resveratrol was loaded into the magnetic polymer by stirring at 100 rpm a 25 °C for 20 min. Then, HP-β-CD-EPI-MN polymer was separated from the solution by magnetic decantation. All the *t*-resveratrol was absorbed into the magnetic polymer since non-residual *t*-resveratrol in the solution was found. After this, the HP-β-CD-EPI-MN polymer loaded with *t*-resveratrol was extracted with 10 mL of ethyl acetate in continuous stirring using a magnetic stirrer. The desorption experiment was maintained for 120 min, taking samples periodically with a micropipette until all the *t*-resveratrol was extracted by the ethyl acetate from HP-β-CD-EPI-MN polymer. The levels of *t*-resveratrol extracted in these samples were analysed by HPLC-DAD, as described by Belchí-Navarro et al. [6]. The experiments were carried out in triplicate.

### 2.7. Statistical Analysis

For each experiment, three independent biological replicates were used. Statistical analysis was performed using the SPSS package (SPSS Inc., Chicago, IL, USA) version 22.0. A two-way analysis of variance (ANOVA) following by Tukey’s HSD post-hoc test was carried out. *p* values < 0.05 were considered as statistically significant.

## 3. Results and Discussion

### 3.1. FE-SEM Characterisation and t-Resveratrol Release Test

**Figure 2:**
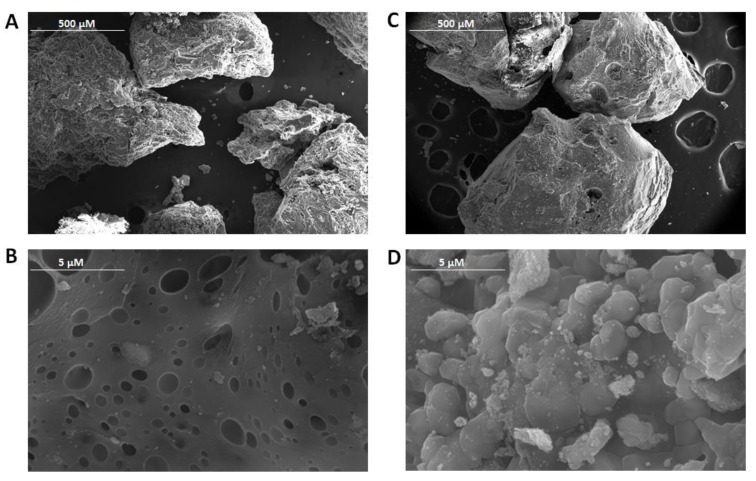
Surface morphology and structure of hydroxypropyl-β-cyclodextrins-epichlorohydrin (HP-β-CD-EPI-MN) polymer (Magnification: 50 × (**A**) and 8000 × (**B**)) and carboxymethyl-β-cyclodextrins-epichlorohydrin (CM-β-CD-EPI-MN) polymer (Magnification: 50 × (**C**) and 8000 × (**D**)) obtained from field emission scanning electron microscope (FE-SEM) images.

Field emission scanning electron microscopy (FE-SEM) was used to characterize the surface morphology of unloaded CM-β-CD-EPI-MN and HP-β-CD-EPI-MN polymers, as shown in Figure 2. The surface morphologies of the CM-β-CD-EPI-MN and HP-β-CD-EPI-MN polymers have significant differences. In fact, the surface of HP-β-CD-EPI-MN polymer (Figure 2A,B) was compact and showed gaps distributed heterogeneously, while CM-β-CD-EPI-MN polymer (Figure 2C,D) had a surface with aggregates, and did not have a defined shape. Both polymers had an irregular structure, suitable for entrapping *t*-resveratrol. The corresponding EDX spectra were also taken, and are shown in Supplementary Data 1 and 2. Bradruddoza et al. [26] and Yu et al. [27] have extensively characterized these polymers with adsorption studies, and evaluated the efficiency of the polymers under different parametric values, such as contact time, adsorbent dosage, initial dye concentration, pH of initial solution and temperature, amongst others.

**Figure 3:**
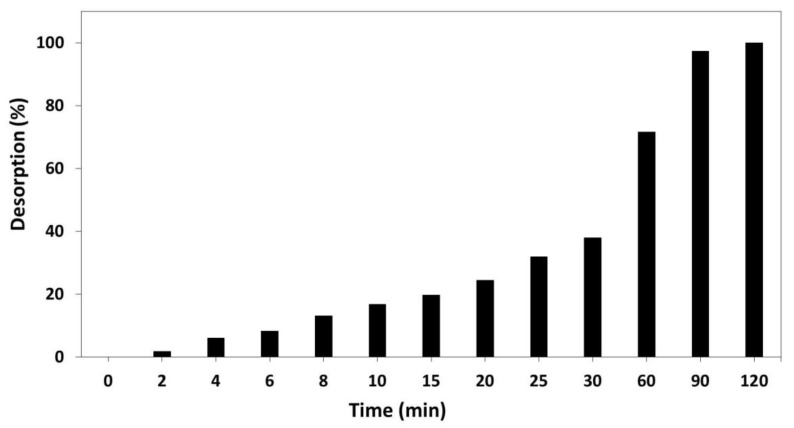
Extraction time course of *t*-resveratrol in ethyl acetate. The desorption process was expressed as the percentage of *t*-resveratrol recovered in ethyl acetate at each time analysed.

In order to analyse the absorption-desorption process of *t*-resveratrol, 150 mg of HP-β-CD-EPI-MN polymer was loaded with commercial *t*-resveratrol at a final concentration of 50 mg/L (219 µM) by stirring for 20 min at 100 rpm (25 °C), as described in Materials and Methods. After this, no residual *t*-resveratrol was detected in the aqueous solution. *t*-Resveratrol trapped in HP-β-CD-EPI-MN polymers was extracted with ethyl acetate in continuous stirring by using a magnetic stirrer. The release of *t*-resveratrol from the HP-β-CD-EPI-MN polymer was continuous, observing that all the *t*-resveratrol was extracted from the polymer after 120 min of extraction with ethyl acetate (Figure 3).

As shown in Figure 3, a 37.92% and 71.57% of *t*-resveratrol was released from HP-β-CD-EPI-MN polymer after 30 and 60 min of extraction, respectively. Moreover, after 120 min of extraction with ethyl acetate, all *t*-resveratrol contained within the polymer had been released, indicating that this extraction time is sufficient to recover all *t*-resveratrol from polymer. Lungoci et al. [22] also observed a continuous release of protocatechuic acid for 72 h using magnetic anionic sulfobutylether-β-CDs polymer loaded with protocatechuic acid.

### 3.2. Effect of Different Concentrations of CM-β-CD-EPI-MN or HP-β-CD-EPI-MN Polymers in the Presence of 100 µM MJ on t-Resveratrol Production in V. vinifera Suspension-Cultured Cells

**Figure 4:**
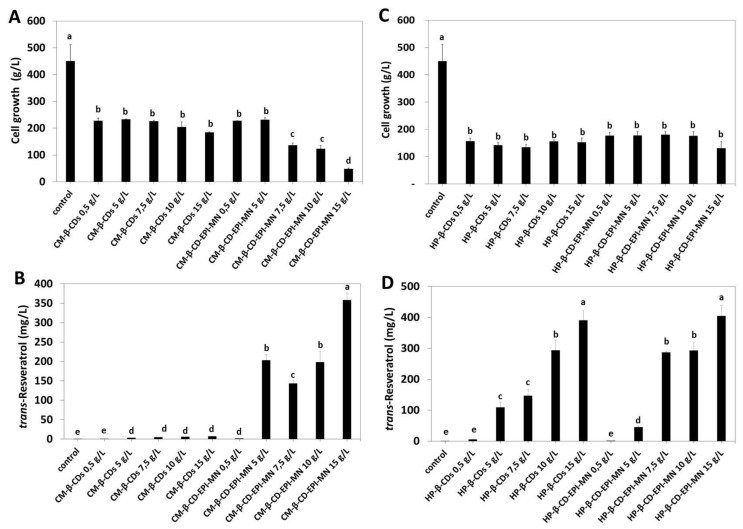
(**A**) Effect of different concentrations of carboxymethyl-β-cyclodextrins-epichlorohydrin (CM-β-CD-EPI-MN) polymer in combination with 100 µM methyl jasmonate (MJ) on cell growth of *Vitis vinifera* suspension-cultured cells treated for 144 h. (**B**) Effect of different concentrations of carboxymethyl-β-cyclodextrins-epichlorohydrin (CM-β-CD-EPI-MN) polymer in combination with 100 µM methyl jasmonate (MJ) on extracellular *t*-resveratrol production in *Vitis vinifera* suspension-cultured cells. (**C**) Effect of different concentrations of hydroxypropyl-β-cyclodextrins-epichlorohydrin (HP-β-CD-EPI-MN) polymer in combination with 100 µM methyl jasmonate (MJ) on cell growth of *Vitis vinifera* suspension-cultured cells treated for 144 h. (**D**) Effect of different concentrations of hydroxypropyl-β-cyclodextrins-epichlorohydrin (HP-β-CD-EPI-MN) polymer in combination with 100 µM methyl jasmonate (MJ) on extracellular *t*-resveratrol production in *Vitis vinifera* suspension-cultured cells. Values are given as the mean ± SD of three experiments with three replicates. Bars with different letters within each time denote statistically significant differences according to Tukey’s test (*p* < 0.05).

The effect of free β-CD or CM-β-CD-EPI-MN or HP-β-CD-EPI-MN polymers on cell growth of *V. vinifera* suspension-cultured cells was checked by determining the final cell fresh weight (expressed as g/L) at 144 h of elicitation (Figure 4A,C). As shown in Figure 4A, the addition of different concentrations of free CM-β-CDs or CM-β-CD-EPI-MN polymer (from 0.5 to 15 g/L) in combination with 100 µM MJ to *V. vinifera* suspension-cultured cells decreased the cell growth, with a further decrease when these suspension-cultured cells were treated with 7.5, 10 and 15 g/L CM-β-CD-EPI-MN polymer and 100 µM MJ. In fact, a decrease of 70% and 90% in cell growth was found when 10 and 15 g/L CM-β-CD-EPI-MN polymers and 100 µM MJ were added to *V. vinifera* suspension-cultured cells, respectively. Likewise, free HP-β-CDs or HP-β-CD-EPI-MN polymer (from 0.5 to 15 g/L) in combination with 100 µM MJ also decreased the cell growth, but to a lesser extent than in cells treated with CM-β-CD-EPI-MN polymer (Figure 4A,C). In fact, the final cell growth was of 48 and 170.88 g cell FW/L in the presence of 15 g/L CM-β-CD-EPI-MN and 15 g/L HP-β-CD-EPI-MN polymers, respectively.

**Figure 5:**
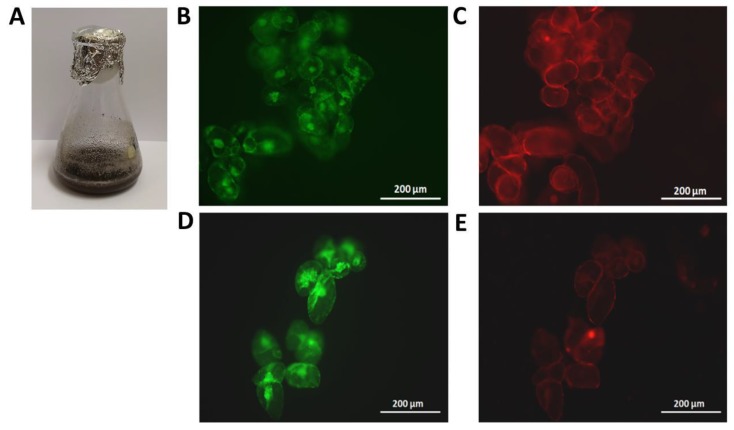
Cell viability (assessed with fluorescein diacetate and propidium iodide as described in Materials and Methods). (**A**) Cell growth observed in a flask containing *V. vinifera* suspension-cultured cells treated with 15 g/L carboxymethyl-β-cyclodextrins-epichlorohydrin (CM-CDs-EPI-MN) + methyl jasmonate (MJ). (**B**) *Vitis vinifera* cells (which have been elicited with 15 g/L carboxymethyl-β-cyclodextrins-epichlorohydrin (CM-CDs-EPI-MN) + 100 µM methyl jasmonate (MJ)) treated with fluorescein diacetate probe. (**C**) *Vitis vinifera* cells (which have been elicited with 15 g/L CM-CDs-EPI-MN + 100 µM MJ) treated with propidium iodide probe. (**D**) *Vitis vinifera* cells (which have been elicited with 15 g/L hydroxypropyl-β-cyclodextrins-epichlorohydrin (HP-CDs-EPI-MN) + 100 µM methyl jasmonate (MJ)) treated with fluorescein diacetate probe. (**E**) *Vitis vinifera* cells (which have been elicited with 15 g/L hydroxypropyl-β-cyclodextrins-epichlorohydrin (HP-CDs-EPI-MN) + 100 µM methyl jasmonate (MJ)) treated with propidium iodide probe. All images were taken at 144 h of treatment.

Although cell growth was stopped in elicited *V. vinifera* suspension-cultured cells, cell viability seems to be little affected compared to control cells, as assessed by fluorescence microscopy using fluorescein diacetate and propidium iodide probes in treated cells (Figure 5). Our results agreed with those found by Belchí-Navarro et al. [6], who found that the addition of 65.5 g/L randomly dimethylated-β-CDs and 100 µM MJ to *V. vinifera* cv Monastrell suspension-cultured cells led to a decrease in cell growth, showing a reduction of 60% at 96 h of treatment. Donnez et al. [28] also observed an inhibition of cell growth of *V. vinifera* cv Chasselas and *Vitis berlandieri* suspension-cultured cells in the presence of MJ. Moreover, Vidal-Limon et al. [29] found that the final biomass levels were lowest in *Vitis vinifera* suspension-cultured cells treated with β-CDs+MJ separately or in combination with hexenol than in control cells after 168 h of treatment. This effect of elicitors on cell growth could be attributed to a change in the metabolic flux, which increases the activation of the secondary metabolism, such as the production of *t*-resveratrol, and decreases processes of the primary metabolism, such as cell division and growth [15,30].

As regards *t*-resveratrol production, the elicitation with free CM-β-CDs and 100 µM MJ (Figure 4B) induced a slight increase in the levels of this compound under the best conditions (6.71 ± 0.53 mg/L). However, when *V. vinifera* suspension-cultured cells were elicited with CM-β-CD-EPI-MN polymer and 100 µM MJ, an increase was detected, resulting in the maximal levels of *t*-resveratrol in the presence of 15 g/L CM-β-CD-EPI-MN polymer (357.95 ± 18.46 mg/L) at 144 h of treatment (Figure 4B). Therefore, under the same elicitation conditions, the production of *t*-resveratrol was about 54 times higher in CM-β-CD-EPI-MN polymer-treated cells than those treated with free CM-β-CDs (Figure 4B).

Furthermore, the effect of different concentrations of free HP-β-CDs or HP-β-CD-EPI-MN polymer in combination with 100 µM MJ on *t*-resveratrol production by *V. vinifera* suspension-cultured cells during 144 h was studied. As shown in Figure 4D, the maximum level of *t*-resveratrol produced and secreted by cells to the extracellular medium was detected in the presence of 15 g/L free HP-β-CDs or 15 g/L HP-β-CD-EPI-MN polymer in combination with 100 µM MJ (390.53 ± 31.72 and 404.53 ± 34.12 mg/L, respectively) (Figure 4D). The production of *t*-resveratrol was over 78-fold higher in *V. vinifera* cells treated with 15 g/L free HP-β-CDs or 15 g/L HP-β-CD-EPI-MN polymer than when cells were elicited with 0.5 g/L free HP-β-CDs or HP-β-CD-EPI-MN polymer. Moreover, other stilbenes such as *t*-piceid or ε-viniferin were not detected in the HP-β-CD-EPI-MN polymer or in the extracellular medium in any samples analysed by HPLC-DAD (Supplementary Data 3).

In recent decades, several studies have been focused on increasing the production of *t*-resveratrol by using different elicitors. In fact, elicitation is an effective technique to enhance the amount of secondary metabolites in plant in vitro cultures, including *t*-resveratrol [31]. Thus, Santamaria et al. [32] observed that jasmonic acid (1.55 mg/L) and MJ (0.27 mg/L) increased extracellular *t*-resveratrol production in *V. vinifera* cv Italia cell cultures. Moreover, Belchí-Navarro et al. [6] found that the production of *t*-resveratrol in *V. vinifera* cv Monastrell suspension-cultured cells was increased in the presence of randomly dimethylated-β-CDs alone (737.0 mg/L). MJ was also the most effective elicitor for enhancing *t*-resveratrol production (60 mg/L) in *V. vinifera* cv. Negramaro [33], but the use of 12-oxo-phytodienoic acid, jasmonic acid, or coronatine, improved the production of viniferins in these cell cultures (200 mg/L). Xu et al. [34] also studied the combined treatment of salicylic acid and UV-C light in *V. vinifera* L. cv. Cabernet Sauvignon suspension-cultured cells, and they observed an increase in the extracellular *t*-resveratrol production under elicitation conditions (2.33 mg/L). In a similar way, when 28 mg/L ethephon and a hydrostatic pressure of 40 MPa were jointly used as elicitors in *V. vinifera* cv Gamay Fréaux suspension-cultured cells, an increase in *cis*-piceid production (0.56 mg/L) was detected compared to control cells [35].

Although this is the first time that CD polymers have been used in living cell systems, there are some works describing the use of these CD polymers as efficient adsorbents of plant metabolites from solutions or acting as nanocarriers in non-living systems [22,36,37,38]. In this way, Lungoci et al. [22] obtained nanocarriers from magnetite nanoparticles, which formed inclusion complexes between anionic sulfobutylether-β-CDs and protocatechuic acid for active drug delivery, proving that this procedure was a good strategy to load and transport active principles that were unstable and insoluble in water. Li et al. [39] also showed that β-CDs functionalized the magnetic-reduced graphene oxide composite, and they were successfully applied for selective extraction and determination of naphthalene-derived phytohormones (1-naphthalene acetic acid and 2-naphthoxyacetic acid) in complex matrices obtained from fresh tomatoes. Moreover, Gong et al. [23] used CM-HP-β-CD polymers modified with magnetic particles to extract in solid phase rutin, a flavonoid glycoside able to increase the resistance of the varicose veins, and dissolve the fat invading the liver. The maximum adsorption capacity for rutin was 67.0 mg/g after 30 min of incubation at room temperature [23]. What is more, a novel smart thermo-responsive-magnetic polymer nanocomposite was prepared using acryl functionalized β-CDs, N-isopropylacrylamide and Fe_3_O_4_ as functional monomers, and curcumin as target molecule. The results of the adsorption experiments showed that these polymers had high selectivity, affinity and adsorption capacity for curcumin. In addition, other benefit of these polymers was their magnetic properties which facilitated the separation process with the help of an external magnet [37].

In addition, Liu et al. [40] designed an organic zinc-metal magnetic network functionalized with β-CDs. The polymer was used as a magnetic porous adsorbent in order to extract triazole fungicides and prochloraz from samples of tomato and lettuce.

All these results indicated that CD polymers were able to form inclusion complexes with plant metabolites, and in this work it has also been demonstrated that CD polymers are not only able to extract metabolites from the culture medium, they are also able to induce their production.

### 3.3. Effect of Elicitation Time Course on t-Resveratrol Production in V. vinifera Suspension-Cultured Cells Treated with 15 g/L HP-β-CD-EPI-MN Polymer and 100 µM MJ

**Figure 6:**
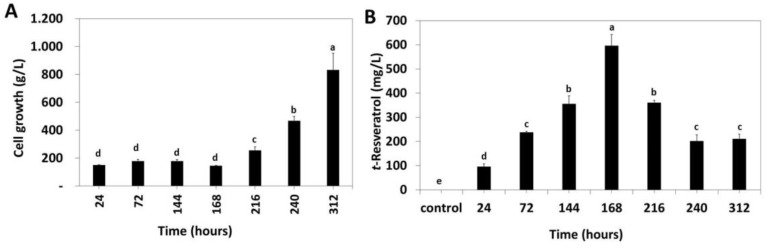
Effect of elicitation time on both cell growth (**A**) and *t*-resveratrol production (**B**) in *V. vinifera* suspension-cultured cells treated with 15 g/L hydroxypropyl-β-cyclodextrins-epichlorohydrin (HP-β-CD-EPI-MN) polymer in combination with 100 µM methyl jasmonate (MJ). Values are given as the mean ± SD of three experiments with three replicates. Bars with different letters within each time denote statistically significant differences according to Tukey’s test (*p* < 0.05).

Taking into account that when 15 g/L CM-β-CD-EPI-MN and 15 g/L HP-β-CD-EPI-MN polymers were used, the final levels of *t*-resveratrol were similar but cell growth was less affected by HP-β-CD-EPI-MN, this last polymer was selected in order to study the effect of the elicitation time course on both cell growth and *t*-resveratrol production. As shown in Figure 6A, the final FW (at 312 h) in *V. vinifera* suspension-cultured cells treated with HP-β-CD-EPI-MN polymer and MJ (832.16 ± 120.51 g/L) increased (around 18%), in comparison with the initial conditions (at 0 time).

In addition, the presence of HP-β-CD-EPI-MN and MJ inhibited cell growth for the first 168 h of elicitation (Figure 6A) that corresponds to the increase in *t*-resveratrol production (Figure 6B), suggesting that, in this period, the activation of the secondary metabolism (production of *t*-resveratrol) is favoured over the primary metabolism (cell division and growth).

Furthermore, the time course of *t*-resveratrol production in *V. vinifera* suspension-cultured cells elicited with 15 g/L HP-β-CD-EPI-MN polymer and 100 µM MJ was analysed (Figure 6B). As can be observed in Figure 6B, a continuous extracellular *t*-resveratrol production was observed from 24 to 168 h of elicitation. The highest levels of *t*-resveratrol were detected at 168 h of treatment (595.96 ± 46.39 mg/L), decreasing these levels until 210 ± 19.91 mg/L at 312 h of elicitation. In addition, *trans*-resveratrol was not detected in either the control or the extracellular media, at any times analysed. The results obtained by Belchí-Navarro et al. [6] were in accordance with those obtained in the presence of 15 g/L HP-β-CD-EPI-MN polymer and 100 µM of MJ (Figure 6B), since these authors showed that the maximal levels of *t*-resveratrol obtained in *V. vinifera* suspension-cultured cells treated with 65.5 g/L randomly dimethylated-β-CDs and 100 µM of MJ were detected at 168 h of elicitation. Similarly, the combination of 1 µM coronatine and 65.5 g/L β-CDs induced the maximal levels of *t*-resveratrol at 168 h of treatment in *V. vinifera* suspension-cultured cells [15]. All these results showed that both HP-β-CD-EPI-MN polymer and free β-CDs were able to increase the production levels of *t*-resveratrol in a similar manner, reaching the highest levels at 168 h of treatment.

### 3.4. Regeneration of HP-β-CD-EPI-MN Polymer

**Figure 7:**
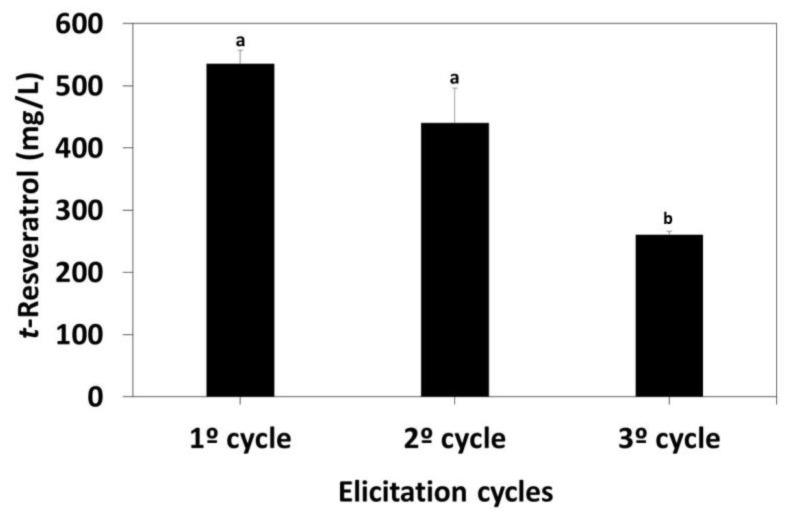
Variation in *t*-resveratrol production through three continuous elicitation cycles (168 h each) using the same hydroxypropyl-β-cyclodextrins-epichlorohydrin (HP-β-CD-EPI-MN) polymer but changing grapevine cells which, in turn, were elicited with the recovered hydroxypropyl-β-cyclodextrins-epichlorohydrin (HP-β-CD-EPI-MN) polymer in combination with 100 µM methyl jasmonate (MJ). Values are given as the mean ± SD of three experiments with three replicates. Bars with different letters within each time denote statistically significant differences according to Tukey´s test (*p* < 0.05).

The potential reusability of HP-β-CD-EPI-MN is essential for their use as an elicitor with long-term durability, and to achieve an effective reduction in the polymer recovery costs. In this sense, effectiveness of reusability was assessed by means of the analysis of the results obtained from three repeated cycles of elicitation using the same HP-β-CD-EPI-MN polymer and 100 µM of MJ, and changing the *V. vinifera* suspension-cultured cells. After each elicitation cycle, the polymer was magnetically separated and recovered from the suspension-cultured cells, and then the recovered HP-β-CD-EPI-MN polymer was incubated with ethyl acetate for 45 min (three times) in order to extract *t*-resveratrol, and then to quantify its levels. As shown in Figure 7, the production of *t*-resveratrol was of 534.90 ± 22.04 mg/L in the first cycle of elicitation.

The recovered polymer was reused in a second cycle of elicitation, observing a final *t*-resveratrol concentration of 439.74 ± 56.34 mg/L, indicating that this polymer retained 82% of its adsorption capacity after two cycles of elicitation; therefore, it is significant to be reused. Once again, polymer was recovered and reused in a third cycle of elicitation, but levels of *t*-resveratrol (260 ± 5.92 mg/L) were lower than those observed in the previous cycles. The capability of induction and adsorption of HP-β-CD-EPI-MN polymer after three cycles of elicitation was of 45% in comparison with the first cycle of elicitation (Figure 7). In this sense, Ragavan and Rastogi [41] synthesized β-CDs capped with graphene-magnetite nanocomposites, and they used them as adsorbents for extracting bisphenol-A in water. These authors observed that regenerated nanocomposites could be reused for the adsorption of bisphenol-A, and they detected that these nanocomposites retained 80% of their adsorption capacity after six cycles of reuse. Moreover, Liu et al. [40] carried out experiments of desorption and regeneration to investigate the recyclability of the magnetic CD porous polymer. They observed that the removal efficiency of organic pollutants remained at 86.35% even after seven cycles. Hence, the results indicated that magnetic CDs porous polymer not only exhibited higher adsorption capacity for organic pollutants in waste water, but also had recyclability, and therefore could be used for practical purposes.

## 4. Conclusions

Different strategies have been carried out in order to raise the levels of *t*-resveratrol in plant in vitro cultures and try to produce it on an industrial scale. One of the most effective strategies to increase its productivity in a short period of time has been the use of CDs as elicitors. However, the price of CDs is high, and therefore it increases the production costs of *t*-resveratrol, making its industrial exploitation economically unfeasible. In this work, two new polymers of CM-β-CD-EPI-MN and HP-β-CD-EPI-MN have been synthesized and, jointly with MJ, were added to *V. vinifera* suspension-cultured cells for inducing and adsorbing selectively *t*-resveratrol, so that these CD polymers could be reused. The results obtained from FE-SEM showed that the surface morphologies of CM-β-CD-EPI-MN and HP-β-CD-EPI-MN polymers had significant differences. In fact, the surface of the HP-β-CD-EPI-MN polymer was compact and showed gaps that were distributed heterogeneously, while CM-β-CD-EPI-MN polymers had a surface with aggregates, and did not have a defined shape. Moreover, the results also indicated that 120 min is the optimal extraction time to recover all *t*-resveratrol from polymers in elicited *V. vinifera* suspension-cultured cells.

On the other hand, elicitation experiments indicated that the production of *t*-resveratrol in the presence of free CM-β-CDs was much lower than those found in the presence of the CM-β-CD-EPI-MN polymer. In addition, the production of this stilbene was enhanced when *V. vinifera* suspension-cultured cells were treated with HP-CDs-EPI polymers, and the decrease in cell growth was less drastic when this polymer was used. Therefore, HP-CDs-EPI polymer was selected in order to optimize the elicitation time for the production of *t*-resveratrol from this living cell system. Moreover, maximal levels of *t*-resveratrol were found at 168 h of elicitation in the presence of the HP-β-CD-EPI-MN polymer and MJ, and no *t*-resveratrol was found in the extracellular medium, indicating that all *t*-resveratrol produced by the cells and secreted into the culture medium was trapped and then recovered by the polymers. Other benefits of using CD polymers were their magnetic properties, which facilitated their separation process with the help of an external magnet. Finally, these results showed that CD polymers can be reused during three cycles of elicitation, since the induction and adsorption capacity of HP-β-CD-EPI-MN polymer after these three cycles remained high, allowing to achieve high concentrations of *t*-resveratrol. Therefore, using HP-CDs-EPI and MJ polymers as elicitors, we have designed a high *t*-resveratrol production system, which reduces production costs since these new polymers can be reused up to three times.

## Supplementary Materials

The following are available online at https://www.mdpi.com/2073-4360/12/4/991/s1.
