# Supplementary material for: A Smart Strategy to Improve t-Resveratrol Production in Grapevine Cells Treated with Cyclodextrin Polymers Coated with Magnetic Nanoparticles
Source: Polymers (Basel). 2020 Apr 24;12(4):991. doi: 10.3390/polym12040991 (PMC7240392; doi:10.3390/polym12040991)
Supplement: Supplementary file 1 [file polymers-12-00991-s001.pdf]

polymers

MDPI

SUPPLEMENTARY MATERIAL

A Smart Strategy to Improve *t*-resveratrol Production in Grapevine Cells Treated with Cyclodextrin Polymers Coated with Magnetic Nanoparticles

Lorena Almagro [1,*], Alicia De Gea-Abellán [1], María Isabel Rodríguez-López [2], Estrella Núñez-Delicado [2], José Antonio Gabaldón [2] and María Angeles Pedreño [1]

[1] Departamento de Biología Vegetal, Facultad de Biología, Universidad de Murcia, Campus de Espinardo, E-30100 Murcia, Spain; aliciadegeaabellan@gmail.com (A.D.G.-A.); mpedreno@um.es (M.A.P.)
[2] Departamento de Tecnología de la Alimentación y Nutrición, Universidad Católica San Antonio de Murcia. Campus de los Jerónimos, E-30107, Murcia, Spain; mirodriguez@ucam.edu (M.I.R.-L.); enunez@ucam.edu (E.N.-D.); jagabaldon@ucam.edu (J.A.G.)

* Correspondence: lorena.almagro@um.es, Tel: +34 868884904

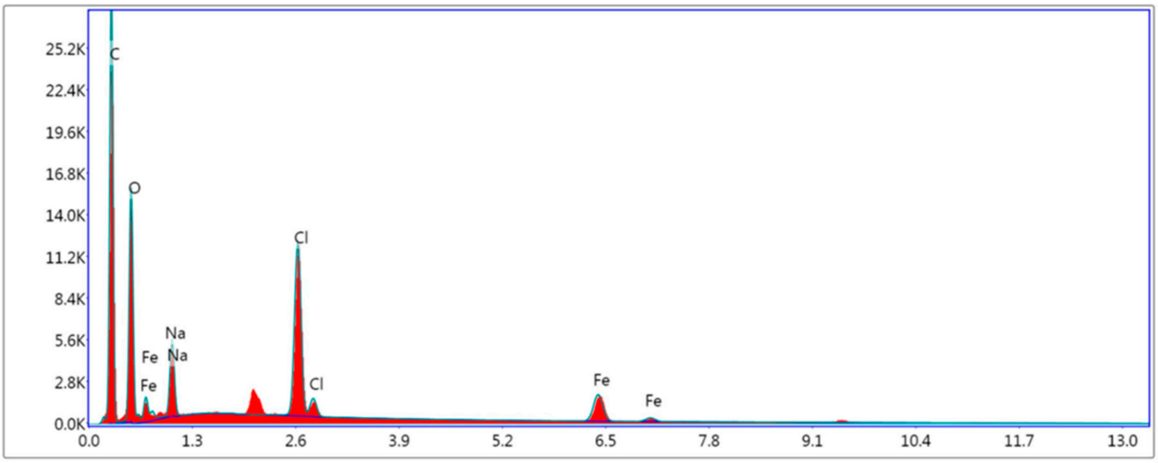

Element	Weight %	Atomic %	Net Int.	Error %	Kratio	Z	R	A	F
C K	55.31	66.17	4,594.03	7.92	0.19	1.04	0.98	0.32	1
O K	31.35	28.16	2,946.88	9.63	0.05	0.99	1	0.16	1
NaK	3.85	2.40	1,108.17	8.01	0.01	0.9	1.02	0.37	1
ClK	5.56	2.25	3,684.56	1.54	0.05	0.84	1.06	0.98	1
FeK	3.94	1.01	843.53	2.76	0.03	0.76	1.09	1.02	1

Fig. S1. EDX spectra of the different carboxymethyl-β-cyclodextrins-epichlorohydrin (CM-CDs-EPI-MN) polymers.

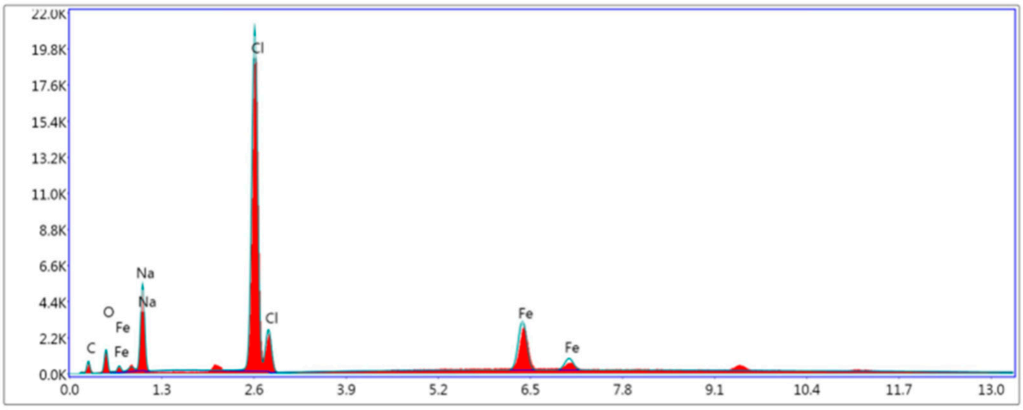

Element	Weight %	Atomic %	Net Int.	Error %	Kratio	Z	R	A	F
C K	16.78	34.31	113.18	12.30	0.02	1.16	0.92	0.09	1
O K	8.89	13.65	261.34	10.70	0.02	1.12	0.94	0.17	1
NaK	16.46	17.58	1,229.46	8.39	0.05	1.02	0.97	0.32	1
ClK	35.63	24.68	6,602.75	2.25	0.31	0.95	1.01	0.9	1.01
FeK	22.24	9.78	1,415.21	2.18	0.19	0.86	1.06	0.99	1

Fig. S2. EDX spectra of the different hydroxypropyl-β-cyclodextrins-epichlorohydrin (HP-CDs-EPI-MN) polymers.

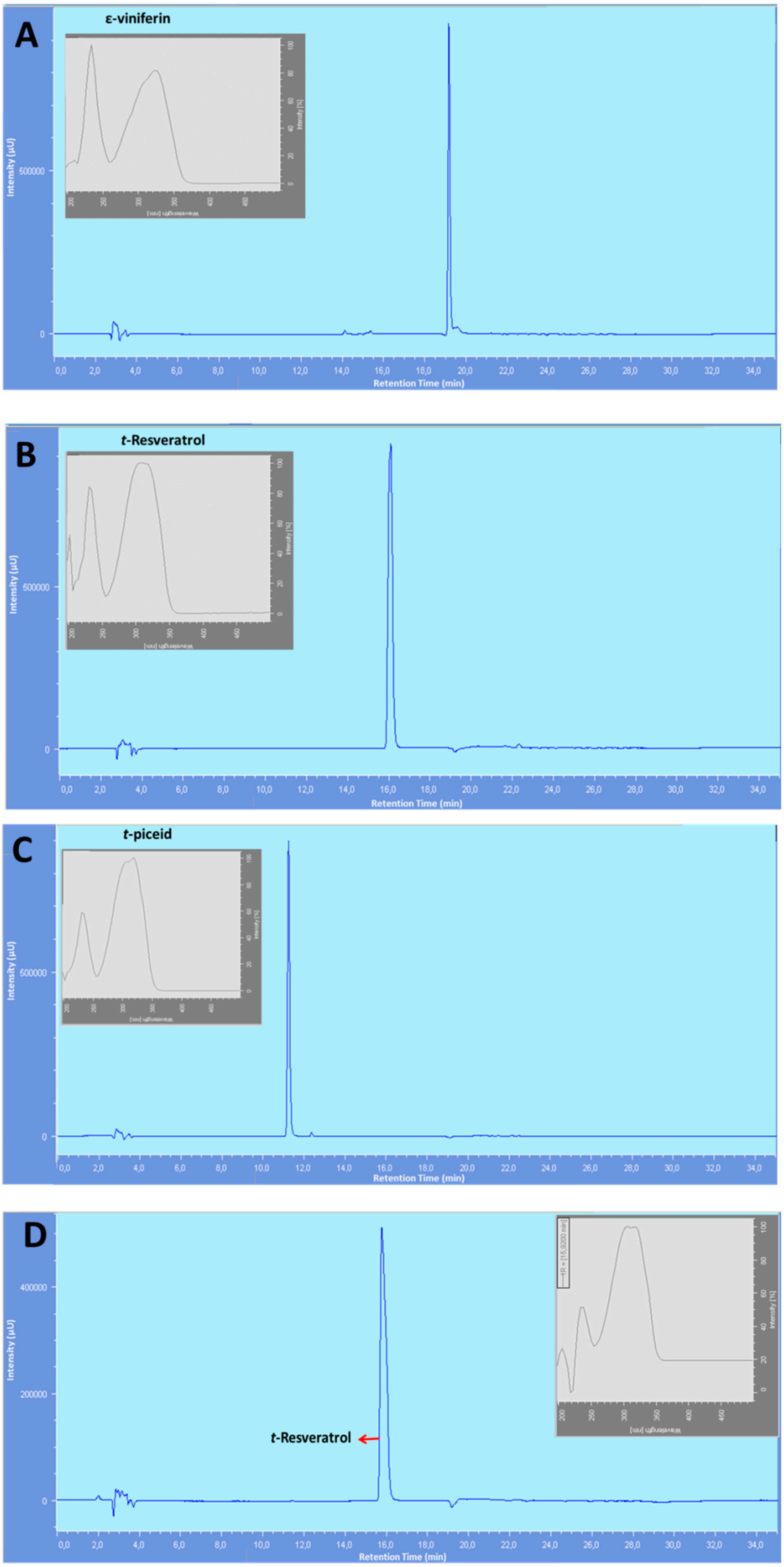

Fig. S3. HPLC-DAD chromatogram of A) ε-viniferin; B) *t*-resveratrol and C) *t*-piceid at 325, 306 and 317 nm, respectively. D) Chromatogram of the sample obtained from hydroxypropyl-β-cyclodextrins-epichlorohydrin (HP-CDs-EPI-MN) extracted with ethyl acetate after the elicitation of *Vitis vinifera* suspension-cultured cells with 100 μM methyl jasmonate and 15 g/L hydroxypropyl-β-cyclodextrins-epichlorohydrin (HP-CDs-EPI-MN) polymers.
